# Pericarditis Caused by *Enterococcus faecium* with Acute Liver Failure Treated by a Multifaceted Approach including Antimicrobials and Hemoadsorption

**DOI:** 10.1155/2021/8824050

**Published:** 2021-03-16

**Authors:** Thomas Köhler, Mathias W. Pletz, Simon Altmann, Carmen Kirchner, Elke Schwier, Dietrich Henzler, Günther Winde, Claas Eickmeyer

**Affiliations:** ^1^Department of Anesthesiology, Surgical Intensive Care, Emergency and Pain Medicine, Ruhr University Bochum, Klinikum Herford, Herford, Germany; ^2^Institute for Infectious Diseases and Infection Control, Jena University Hospital, Jena, Germany; ^3^Department of General and Visceral Surgery, Thoracic Surgery and Proctology, Ruhr University Bochum, Klinikum Herford, Herford, Germany

## Abstract

**Background:**

Sepsis and septic shock are still life-threatening diseases with a high mortality rate. We report a complex case of peritonitis with pericarditis and acute liver failure caused by septic shock. Potentially hepatotoxic antibiotic therapy levels were monitored using the liver maximum capacity (LiMAx®) test, and standard treatment was supplemented by adjunctive hemoadsorption with CytoSorb®. *Case Presentation.* The case features a 29-year-old woman with a history of Crohn's disease and cachexia. Peritonitis caused by *Enterococcus faecium* was diagnosed later due to an ileum perforation. The hematogenic spread led to pericarditis. In addition, sepsis-related acute liver failure complicated antimicrobial therapy further. The combination of standard therapy, anti-infective medication, and blood purification was associated with inflammation control, hemodynamic stabilization, and a concomitant decrease in vasopressor support. An efficient, sustained reduction in plasma bilirubin levels was achieved while maintaining liver function.

**Conclusions:**

This case shows how complex infectious diseases with an atypical infectious focus resulting in septic shock can be successfully treated. A combination of antimicrobial (tigecycline and caspofungin) and long-term adjunctive hemoadsorption therapy was administered while hepatotoxic antibiotic medication was monitored by liver function testing.

## 1. Background

In 2016, out of a total of 2.94 million German patients with a “cardiac diagnosis,” only 2531 cases were hospitalized due to acute pericarditis (an estimated 0.086% of all cardiac diagnoses) [1]. The most common form of acute pericarditis is viral in origin, self-limiting, and minimally life-threatening. However, bacterial pericarditis differs markedly from that of viral origin and reaches 100% lethality if untreated [2].

Pericarditis represents an inflammatory cardiac disease and can potentially induce systemic hyperinflammation mediated by inflammatory mediators such as IL-1 [3]. In this context, multiorgan failure may occur, further worsening the prognosis. The liver, as an accelerator but also as a victim of the systemic inflammatory process, plays a key role [4]. This must be taken into account particularly when a potentially liver toxic drug (e.g., antibiotics/antifungals) is used.

These rare cases require individual and patient-centered treatment plans. In the present case, we have combined established standard care, including antimicrobial therapy, with new therapeutic modalities such as hemoadsorption with CytoSorb® and LiMAx. CytoSorb® therapy is used for elevated cytokine and bilirubin levels. When combined with the liver maximum capacity test (LiMAx), an equally innovative diagnostic tool to adjust for anti-infective medication in the setting of sepsis-associated liver failure, the effects are additive. These concepts are therefore of great value for our daily clinical work and management of complex diseases.

We report here the case of a 29-year-old woman with known Crohn's disease and cachexia who developed bacterial pericarditis caused by *Enterococcus faecium*, most likely based on peritonitis followed by acute liver failure. Our approach included a personalized treatment plan consisting of antimicrobials, hemoadsorption, and liver function tests which lead to a full recovery of the patient (within 13 weeks).

## 2. Case Presentation

A 29-year-old woman with a history of Crohn's disease and cachexia presented with painful diarrhea and unintentional weight loss of 13 kilograms over the past three weeks caused by a mechanical ileus. Transfer to the intensive care unit (ICU) occurred 2 weeks later because of increasing somnolence, impaired gas exchange (PaO2 48 mmHg), and high norepinephrine requirements (1.56 *μ*g/kg/min). Laboratory chemistry revealed significantly altered hepatic and inflammatory parameters (albumin 23.8 g/l, gamma-GT 118 U/l, alkaline phosphatase 142 U/l, cholinesterase 1814 U/l, CRP 194.8 mg/l, and procalcitonin 59.80 *μ*g/l). This corresponded to a SOFA score (Sequential Organ Failure Assessment) of 6.

The severity of the clinical picture demanded exploratory laparotomy for source control. Intraoperatively, a perforation with local peritonitis was found in the lower abdomen. A right hemicolectomy, partial resection of the small bowel, and side-to-side anastomosis were performed. Histology revealed massive chronic inflammation of the terminal ileum, typical of Crohn's disease.

Preoperatively started antibiotic therapy with piperacillin/tazobactam was continued for four days according to the resistogram (*Providencia stuartii*, *Escherichia coli*, and anaerobic bacteria). Blood cultures taken on ICU admission were negative. The patient was treated with differentiated volume and catecholamine therapy. As she still required high doses of norepinephrine, a combination of citrate-anticoagulated continuous renal replacement therapy (CRRT) and a total of 3 adjunctive CytoSorb® hemoadsorption therapy sessions (for a total of 73 h) were applied, resulting in a rapid stabilization of her hemodynamic situation (norepinephrine to 8.3% of the maximum initial dose) and extubation on the day of CytoSorb cessation.

Four days after the operation, the patient's condition started to worsen rapidly with tachycardia, hypotension, and fever up to 39.0°C, as well as diminishing oxygen saturation levels. The patient had to be reintubated and norepinephrine administration had to be initiated for hemodynamic stabilization (1.09 *μ*g/kg/min). Over time, hemodynamics stabilized while diuresis resumed spontaneously and renal replacement therapy could be stopped shortly after.

**Figure 1:**
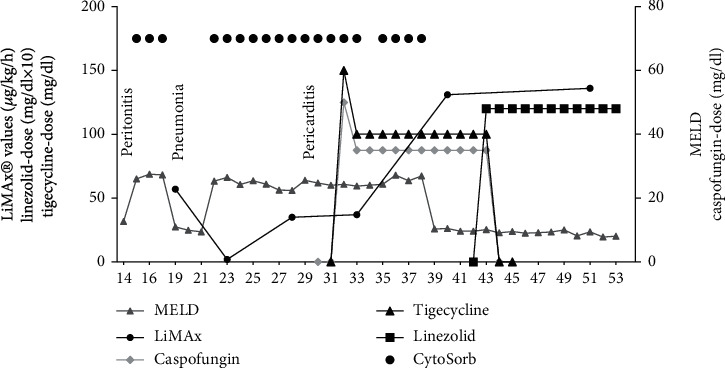
LiMAx® values in comparison to the MELD-Score calculated from conventional liver parameters over the course of treatment and the caspofungin-, tigecycline-, and linezolid-dose administered. Note that CVVHD in combination with CytoSorb® hemoadsorption was restarted at *hospital day* 22. Linezolid dose should be multiplied by a factor of ten for reasons of better representability.

A chest X-ray performed the same day revealed a pleural effusion. In search for an infectious source, samples (pleural fluid, two sets of blood cultures, and bronchoalveolar fluid) were sent to microbiology but proved negative. Abdominal and chest CT scan revealed an intact anastomosis but showed multiple dense foci inside the lungs. Despite the negative microbiology, the decision was made to intensify antibiotic therapy by escalation to meropenem. The dynamic maximum liver function capacity test (LiMAx®) showed a value of 57 *μ*g/kg/h, indicating severe liver insufficiency (Figure 1). In addition, a subsequent CT scan confirmed severe, previously unknown, emphysematic changes (bullae) in the lungs which further compromised gas exchange. Over the next 9 days, both chest X-ray and CT scans indicated morphological improvement. However, the patient's condition rapidly deteriorated again now presenting a multiple organ dysfunction syndrome (SOFA score 12). Inflammatory marker levels were clearly increased and the patient became anuric, while FiO_2_ levels required an increase to 100%. Liver function was still severely impaired. The hypothesis was septic shock syndrome, and blood culture samples were taken; however, neither showed bacterial nor fungal growth.

**Figure 2:**
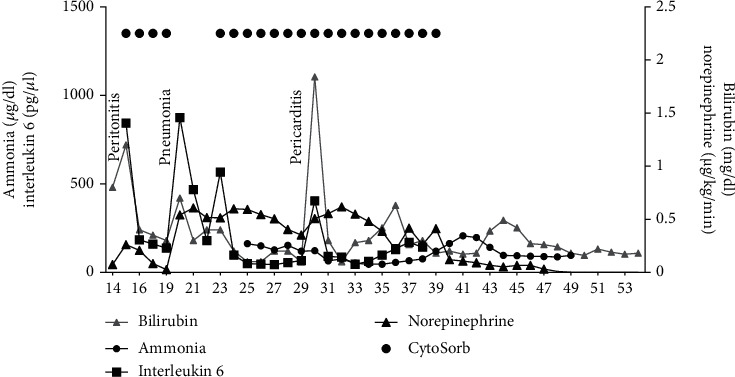
Ammonia, bilirubin, and interleukin 6 serum concentrations as well as norepinephrine dosage over time as well as hemoadsorption with CytoSorb ® starting on hospital day 15 and 22.

As the patient required continuous venovenous hemodialysis (CVVHD) at that point, the decision was made to apply the CytoSorb® system for the second time in order to attenuate the hyperinflammatory response. This was also done to eliminate liver metabolites, such as bilirubin, ammonia, and bile acids in the context of sepsis-associated acute liver failure (Figure 2). Consequently, another 13 CytoSorb adsorber cartridges were applied consecutively for a total duration of 346 hours. With maximum interdisciplinary care (laparotomy, CRRT, liver and ventilation support, pericardial drainage, antimicrobial therapy, etc.), the hyperinflammatory response was declining which resulted in a marked decrease in vasopressor requirements (reduction of vasopressor support to 16.9% of the maximum initial dose, respectively) and a normalization in bilirubin levels (Figure 2) accompanied by the onset of diuresis.

An emergency relaparotomy was performed that excluded any intra-abdominal source of sepsis. However, echocardiography revealed a significant increase in pericardial effusion and pericardial tamponade was diagnosed. The patient underwent a pericardial tap, and a drain was inserted.

**Figure 3:**
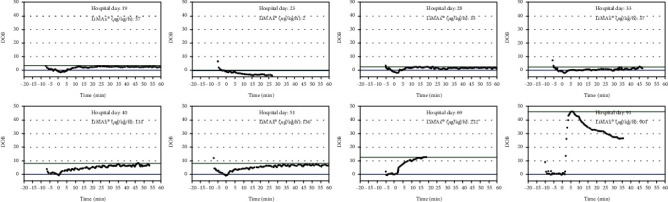
LiMAX® measurement protocols.

One day later, *Enterococcus faecium* was identified in the pericardial fluid but also in all blood cultures, intra-abdominal, and urine and tracheal fluids. All strains proved to have equal resistance patterns suggesting a common origin that was most likely the ruptured ileum. Pathohistologically, a granulocyte-rich pericardial effusion was found to be an expression of a florid inflammation. Based on these findings, an immediate change in the antibiotic regimen was necessary. An antimicrobial chemotherapy supplemented by tigecycline and the antifungal caspofungin was considered appropriate, given the risk profile (parenteral nutrition, intraabdominal perforation, and immunosuppression). Antimicrobial chemotherapy supplemented by caspofungin is potentially hepatotoxic (likelihood score: D (possible cause of clinically detectable liver damage)) [5] and was closely monitored using the LiMAx® test (8 measurements in total). The dose was reduced accordingly (Figure 3) [6]. As for tigecycline, it is not proven to have a negative impact on liver function (likelihood score: E^∗^ (unproven but suspected cause of clinically apparent liver injury)) [7], standard dosing of 50 mg twice daily was applied.

Inflammatory parameters improved consistently over the course of the next week (Figure 2). The catecholamine dose was reduced, and invasive ventilation was changed to assisted ventilation. Gradually, the sedation rate was reduced and the patient regained consciousness. Her mental state improved continually. To facilitate weaning, a tracheotomy was performed. In the meantime, the patient's overall clinical condition improved accompanied by a reduction in SOFA score to 6. As norepinephrine requirements were minimal, CytoSorb® therapy was discontinued. Five days later, *E. faecium* was cultivated from the tip of the central venous catheter under continued antimicrobial therapy with tigecycline. The catheter was removed and tigecycline was changed to linezolid (600 mg twice daily). The LiMAx® measurement another 8 days later showed a stable, but still medium-gross restricted hepatic function (136 *μ*g/kg/h) (Figure 1). After further improvement, the patient was discharged in a stable clinical condition from the ICU to the normal ward 53 days after initial admission.

## 3. Discussion

To the best of our knowledge, this is the first detailed description of an *E. faecium* pericarditis in a patient with complex pathophysiological changes caused by a multitude of different chronic (Crohn's disease, cachexia) and acute diseases (septic shock with multiorgan failure in bacterial pericarditis). The successful treatment was based on a multidisciplinary and multilayered interdisciplinary intervention including anti-infective therapy, hemoadsorption with the CytoSorb®-cartridge, and dynamic liver function testing.

Bacterial, noncardiosurgical pericarditis is very rare [1, 2] and is mainly caused by *Staphylococcus* spp. while *Streptococci*, including *Enterococci*, are much less frequently detected. Apart from an increasing pericardial effusion with life-threatening hemodynamic effects, no clinical symptoms such as chest pain or pericardial rubbing nor ECG alterations or paraclinical findings were detectable in our sedated patient. Echocardiography was the only procedure that quickly led to diagnosis and therapy.

*E. faecium* is a rare pathogen of a very rare acute bacterial pericarditis. It is more likely to occur with immunosuppression [8], seen in our patient who had a high risk of infection due to long-term steroid therapy in combination with an intra-abdominal perforation due to the known Crohn's disease. The most likely cause was a hematogenic scattered infection of the pericardium from the initial intraabdominal focus. Microbiological diagnostics confirmed *Enterococcus faecium* which had caused pericarditis in our patient. We decided to use intravenous therapy with tigecycline in combination with caspofungin, which was dose adapted to the severely impaired liver function. Since there was a risk of insufficient fungicidal caspofungin dosage, monitoring of dynamic liver function with the LiMAx® test was performed in correlation with static liver function values and the clinical presentation with good clinical success.

Patients with septic shock develop liver dysfunction or liver failure [9] and a variety of different drugs commonly used in intensive care medicine (antirheumatics, neuroleptics, antiepileptics, antibiotics, and antifungals) can cause hepatotoxic effects, aggravate a preexisting liver dysfunction, or cause a so-called drug-induced liver failure (DILI) [10–12]. While static liver tests (i.e., bilirubin and transaminases) only provide a snapshot of liver function, the LiMAx® test allows for a quantitative, dynamic measurement of the maximum liver function capacity [13]. To exclude any influence on methacetin metabolism, CVVHD was paused for the duration of each LiMAx® measurement. The LiMAx® results are shown in Figure 1. Note, a result of 315 *μ*g/kg/h or more is physiological [14] and lower values represent a liver function that may be impaired. Any value below 140 *μ*g/kg/h strongly indicates severe liver insufficiency. A test result of less than 100 *μ*g/kg/h in combination with respiratory dysfunction is associated with an increasing mortality rate [15] and values <60 *μ*g/kg/h indicate acute liver failure.

By using the LiMAx® test, we were able to determine the actual functional state of the liver, whereas the standard methods showed a delay of several days between loss of function and paraclinical detection of pathological liver values (Figures 1 and 2). This allowed the detection of a drug-induced impairment of liver function, e.g., by caspofungin, at an early stage and simultaneously time to assess hepatic function in the context of septic multiple organ failure. Hence, instead of the recommended caspofungin dose of 50 mg once daily, we decided to reduce the dose to 35 mg daily, thus possibly preventing further toxic damage. During the course of the clinical improvement, the LiMAx® reading increased to 131 *μ*g/kg/h at hospital day 40 parallel with the clinical improvement as an expression of a slow recovery of liver function under this caspofungin dose, so that no further toxic liver damage was assumed.

Under continuous renal replacement therapy (CRRT), the risk of incorrect or underdosage and therapy failure is increased due to altered pharmacokinetics ([16]; Table 1). Dose recommendations for antimicrobial chemotherapy under CRRT are lacking [17]. The same holds true for the CytoSorb adsorber.

The overall findings in the reported case taking into account the acute threat to life, the microbiological findings, the difficult localisation of the underlying infection, the existing multiorgan failure with severe liver failure, and the need for rapid, bactericidal therapy with immediate achievement of relevant tissue levels led us to intentionally administer an antibiotic therapy with tigecycline in an unchanged standard dose (i.e., without the weight-adapted required dose reduction), under dynamic monitoring of liver function. In cases of severe liver dysfunction, dose adjustment to 2 × 25 mg daily should be performed.

Due to MW > 55 kDa, an interaction with the CytoSorb® adsorber was not very likely. In this regard, relevant adsorption phenomena with extraction rates between 85 and 100% [16] are described for various antibiotics and antifungals (Table 1). Dose adjustment is difficult to extrapolate because clearance is highest within the first two hours [18]. Therefore, the administration of an additional antibiotic/antifungal dose within the first hours should be considered. If possible, an intensive drug monitoring should be carried out. In our case, a relevant adsorption of linezolid by CytoSorb [19, 20] in addition to the described high liver toxicity was a further reason to perform the primary therapy with tigecycline and not with linezolid.

The use of the CytoSorb® adsorber in patients with septic shock is currently the subject of controversial discussion with regard to the right indication, start and duration of therapy, and the potential clinical benefit. We have used CytoSorb® for a previously unpublished total treatment time of 419 h in combined conditions (septic shock/liver failure). During treatment, an interdisciplinary intensive care therapy (laparotomy, CRRT, liver and ventilation support, pericardial drainage, antimicrobial chemotherapy, etc.) in combination with CytoSorb® as an individual adjuvant treatment concept in both cycles (hospital day 15 to 18 and hospital day 22 to 38) allowed to control hyperinflammation and to clearly decrease vasopressor requirements (Figure 2).

Importantly, bilirubin serum concentrations were almost normal throughout the entire course of treatment, which can only be explained by the efficient clearance of CytoSorb over 2.5 weeks of treatment in persistent liver failure (Figure 2). In this regard, recent data shows that the CytoSorb adsorber can dissolve the strong bilirubin-albumin binding and adsorb bilirubin without a relevant change in albumin concentrations [22]. CytoSorb therefore represents a promising, easy to carry out method for liver support. The combination of the adsorption and elimination of bilirubin and bile acids, the modulation of involved cytokines, and the reduction of excess ammonia levels via a parallel renal replacement procedure allows to bridge the time until functional recovery or orthotopic liver transplantation [22]. This concept has been described by several authors [23–25].

## 4. Limitations

In the overall view of the complex treatment process, some aspects remain worth discussing. The antimicrobial treatment with tigecycline and caspofungin for 12 days each followed by linezolid for a total of 41 days (including rehabilitation) led to a full recovery of the patient. Whether a shorter treatment period would have been sufficient is unclear. With regard to the “original” intra-abdominal focus, a possible mixed infection and the existing liver failure, we decided to prioritise tigecycline. Daptomycin is not used for the treatment of pericardial *E. faecium* infection due to its limited spectrum and the ongoing discussion about the optimal dose, although it has a better efficacy compared to linezolid. Furthermore, daptomycin, like linezolid, is classified as toxic to the liver and was therefore not considered here as a therapeutic option (likelihood score: C (probable cause of clinically detectable liver damage). The catheter-associated infection by *E. faecium* at day 43 under ongoing tigecycline medication was possibly caused by the high distribution volume (7-12 l/kg) and the resulting (too) low serum levels (30) [26].

We deliberately decided in favor of echocardiographic monitoring in pericarditis as our risk-benefit analysis and refrained from repeated puncture of the pericardial space. Microbiological sanitation was therefore not detectable.

We assume that the dynamic liver function measurement in septic shock using the LiMAx test reflects the liver function in real time during multiorgan failure. For cost reasons, a more frequent, e.g., daily determination was not possible and thus therapeutically important information might not have been recorded.

Kogelmann et al. recommend changing the CytoSorb cartridges at intervals of 24 hours [27]. In our case, the average changing interval was 30.2 hours. Taking into account the time-dependent saturation kinetics, it must be assumed that the clearance decreases with time. Arguably, a better effectiveness of the hemoadsorption treatment could have been achieved by shortening the lifetime of the CytoSorb® cartridge to 24 hours.

## 5. Conclusions

To the best of our knowledge, this is the first case report of a patient with intra-abdominal perforation caused by Crohn's disease with a secondary pericardial infection which was most likely hematogenically acquired. The patient required a tailored and unconventional treatment approach. In particular, the management of septic liver failure with dynamic liver function test (LiMAx®), adapted antibiotics/antimycotic medication and the long-term use of the CytoSorb® adsorber were described for the first time. Further studies are needed to clarify to what extent the presented concept can contribute to reducing the high mortality in sepsis, which has remained unchanged for decades.

## Figures and Tables

**Table 1:** Drugs for which an absolute or relative drug overdosage/intoxication can be treated with CytoSorb or a relevant decrease of serum concentrations must be expected (in modification of [16, 18–21]).

Drug group	Active pharmaceutical substances adsorbed by CytoSorb
Anticoagulants	Dabigatran, rivaroxaban, ticagrelor
Psychotropic drugs	Quetiapine, venlafaxine, 3,4-methylenedioxymethamphetamine (MDMA, “ecstasy”)
Antiarrhythmics	Flecainide, digoxin
Calcium channel blockers	Amlodipin, verapamil
Anticonvulsants	Carbamazepine, valproic acid, phenytoin
Hypnotics and sedatives	Phenobarbital
Immunosuppressives	Tacrolimus, ciclosporin
Antibiotics	Vancomycin, amikacin, tobramycin, gentamicin, linezolid, teicoplanin, meropenem, imipenem, ciprofloxacin, piperacillin, flucloxacillin
Antimycotics	Voriconazole, fluconazole
Contrast agents	Iodixanol, iohexol
Others	Aflatoxine

## Abbreviations

CRP: C-reactive protein
CRRT: Continuous renal replacement therapy
CVVHD: Continuous venovenous hemofiltration
DILI: Drug-induced liver insufficiency
ICD-10: International Code of Diseases-10
ICU: Intensive care unit
LiMAx: Liver maximum capacity test
MELD: Model for End-Stage Liver Disease
SOFA: Sequential Organ Failure Assessment.
